# 3-Chloro-4-[2-(4-chloro­benzyl­idene)hydrazinyl­idene]-1-methyl-3,4-dihydro-1*H*-2λ^6^,1-benzothia­zine-2,2-dione

**DOI:** 10.1107/S1600536813004443

**Published:** 2013-02-23

**Authors:** Saeed Ahmad, Muhammad Shafiq, M. Nawaz Tahir, William T. A. Harrison, Islam Ullah Khan

**Affiliations:** a Department of Chemistry, Gomal University, Dera Ismail Khan, NWFP, Pakistan; b Department of Chemistry, Government College University, Faisalabad 38000, Pakistan; c Department of Physics, University of Sargodha, Sargodha, Pakistan; d Department of Chemistry, University of Aberdeen, Meston Walk, Aberdeen AB24 3UE, Scotland; e Materials Chemistry Laboratory, Department of Chemistry, Government College University, Lahore, Pakistan

## Abstract

In the title compound, C_16_H_13_Cl_2_N_3_O_2_S, the dihedral angle between the aromatic rings is 6.62 (2)° and the C=N—N=C torsion angle is 176.2 (4)°. The thia­zine ring shows an envelope conformation, with the S atom displaced by 0.633 (6) Å from the mean plane of the other five atoms (r.m.s. deviation = 0.037 Å). The Cl atom is an an axial conformation and is displaced by 2.015 (6) Å from the thia­zine ring plane. In the crystal, mol­ecules are linked by C—H⋯O inter­actions, generating a three-dimensional network. Very weak aromatic π–π stacking inter­actions [centroid–centroid separations = 3.928 (2) Å] are also observed.

## Related literature

For background to benzothia­zines, see: Misu & Togo (2003 ▶); Harmata *et al.* (2006 ▶). For the synthesis and biological activity of the title compound and related materials, see: Ahmad *et al.* (2010*a* ▶); Shafiq *et al.* (2011*a* ▶). For further synthetic details, see: Shafiq *et al.* (2011*b* ▶). For related structures, see: Ahmad *et al.* (2010*b* ▶); Shafiq *et al.* (2011*c* ▶, 2013 ▶).
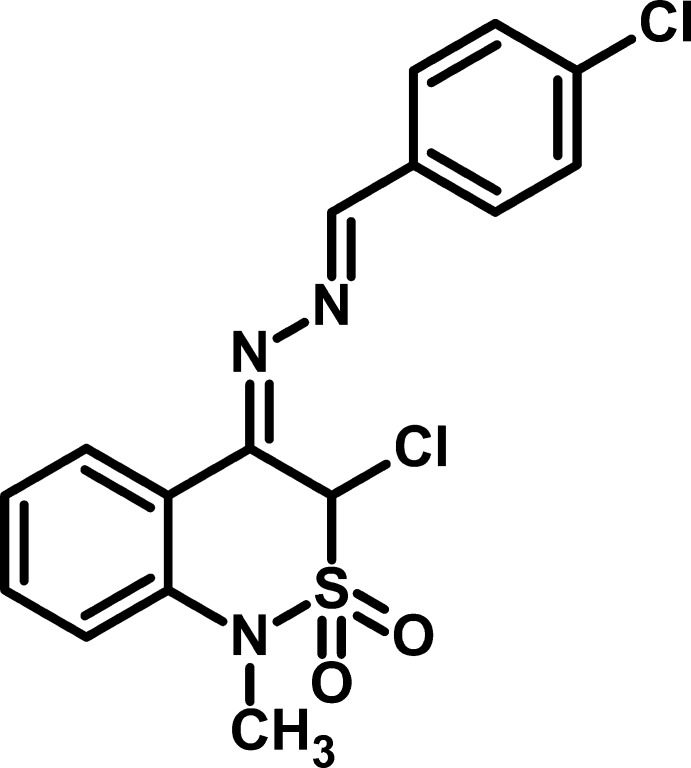


## Experimental

### 

#### Crystal data

C_16_H_13_Cl_2_N_3_O_2_S
*M* *_r_* = 382.25
Monoclinic, 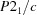

*a* = 12.309 (2) Å
*b* = 17.189 (3) Å
*c* = 8.1837 (13) Å
β = 101.632 (8)°
*V* = 1695.9 (5) Å^3^
*Z* = 4
Mo *K*α radiation
μ = 0.52 mm^−1^
*T* = 296 K
0.28 × 0.16 × 0.14 mm

#### Data collection

Bruker APEXII CCD diffractometer
Absorption correction: multi-scan (*SADABS*; Bruker, 2007 ▶) *T*_min_ = 0.868, *T*_max_ = 0.931
13580 measured reflections
3319 independent reflections
1736 reflections with *I* > 2σ(*I*)
*R*_int_ = 0.076

#### Refinement

*R*[*F* ^2^ > 2σ(*F* ^2^)] = 0.056
*wR*(*F* ^2^) = 0.140
*S* = 1.00
3319 reflections
218 parameters
H-atom parameters constrained
Δρ_max_ = 0.42 e Å^−3^
Δρ_min_ = −0.33 e Å^−3^

### 

Data collection: *APEX2* (Bruker, 2007 ▶); cell refinement: *SAINT* (Bruker, 2007 ▶); data reduction: *SAINT*; program(s) used to solve structure: *SHELXS97* (Sheldrick, 2008 ▶); program(s) used to refine structure: *SHELXL97* (Sheldrick, 2008 ▶); molecular graphics: *ORTEP-3 for Windows* (Farrugia, 2012 ▶); software used to prepare material for publication: *SHELXL97*.

## Supplementary Material

Crystal structure: contains datablock(s) global, I. DOI: 10.1107/S1600536813004443/im2417sup1.cif

Structure factors: contains datablock(s) I. DOI: 10.1107/S1600536813004443/im2417Isup2.hkl

Supplementary material file. DOI: 10.1107/S1600536813004443/im2417Isup3.cml

Additional supplementary materials:  crystallographic information; 3D view; checkCIF report

## Figures and Tables

**Table 1:** Hydrogen-bond geometry (Å, °)

*D*—H⋯*A*	*D*—H	H⋯*A*	*D*⋯*A*	*D*—H⋯*A*
C4—H4⋯O2^i^	0.93	2.48	3.356 (6)	158
C12—H12⋯O2^ii^	0.93	2.59	3.419 (5)	149
C13—H13⋯O1^iii^	0.93	2.50	3.293 (5)	143

Symmetry codes: (i) 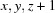; (ii) 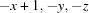; (iii) 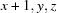.

**Fig. 1.**
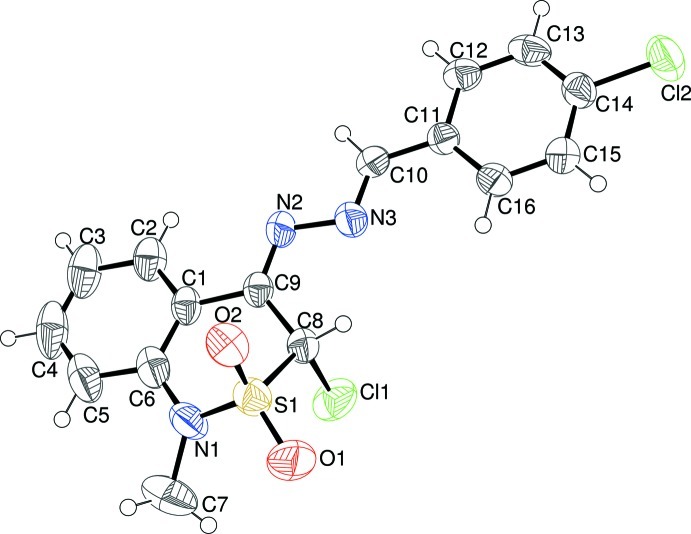
The molecular structure of (I), showing displacement ellipsoids at the 50% probability level.

**Fig. 2.**
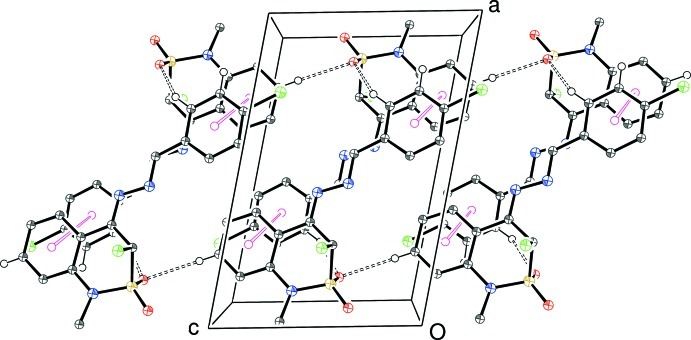
Partial packing diagram of (I), showing H···O interactions as double-dashed lines and π-π stacking as open pink lines.

## supplementary crystallographic information

### Comment

Benzothiazine derivatives are versatile chiral ligands (Harmata *et al.*, 2006) and show various biological activities (Ahmad *et al.*, 2010*a*, Misu & Togo, 2003). As part of our ongoing studies in this area (Shafiq *et al.*, 2011*a*,*b*), we now describe the synthesis and structure of the title compound, (I) (Fig. 1).

The dihedral angle between the aromatic rings C1–C6 and C11–C16 is 6.6 (2)° and the C9=N2—N3=C10 torsion angle is 176.2 (4)°. The conformation of the C1/C6/C8/C9/N1/S1 thiazine ring is an envelope, with the S atom displaced by -0.633 (6) Å from the mean plane of the other five atoms (r.m.s. deviation = 0.037 Å). This displacement is smaller than that seen in related structures (Shafiq *et al.*, 2013). In (I), atom C7 is displaced from the mean plane of the ring by 0.541 (7) Å and Cl1, in an axial site, is displaced by 2.015 (6) Å. Atom C8 is a stereogenic centre with an *S* configuration in the arbitrarily-chosen asymmetric unit. Nevertheless, crystal symmetry indicates a racemic mixture.

In the crystal, moelcules are linked by C—H···O interactions (Table 1) to generate a three-dimensional network (Fig. 2). Very weak aromatic π-π stacking interactions between the benzene rings C1—C6 and C13—C18 benzene rings [centroid-centroid separations = 3.928 (2) Å] are also observed.

### Experimental

The Schiff base derivative of (4*Z*)-4-hydrazinylidene-1-methyl-3,4-dihydro -1*H*-2,1-benzothiazine 2,2-dioxide (Shafiq *et al.*, 2011*c*) and *para*-chlorobenzaldehyde was prepared using the method reported previously (Shafiq *et al.*, 2011*a*). Chlorination of the Schiff base was undertaken using *N*-chloro succinimide and dibenzoylperoxide (Shafiq *et al.*, 2011*b*). The crude product was re-crystallized from ethyl acetate and dichloromethane solution (1:1 v/v) to obtain yellow blocks of the title compound.

### Refinement

H atoms were placed in calculated positions (C—H = 0.93–0.97 Å) and refined as riding. The methyl group was allowed to rotate, but not to tip, to best fit the electron density. The constraint *U*_iso_(H) = 1.2*U*_eq_(C) or 1.5*U*_eq_(methyl C) was applied.

### Crystal data

**Table:**

C_16_H_13_Cl_2_N_3_O_2_S	*F*(000) = 784
*M**_r_* = 382.25	*D*_x_ = 1.497 Mg m^−^^3^
Monoclinic, *P*2_1_/*c*	Mo *K*α radiation, λ = 0.71073 Å
Hall symbol: -P 2ybc	Cell parameters from 225 reflections
*a* = 12.309 (2) Å	θ = 3.7–22.3°
*b* = 17.189 (3) Å	µ = 0.52 mm^−^^1^
*c* = 8.1837 (13) Å	*T* = 296 K
β = 101.632 (8)°	Block, yellow
*V* = 1695.9 (5) Å^3^	0.28 × 0.16 × 0.14 mm
*Z* = 4	

### Data collection

**Table:**

Bruker APEXII CCD diffractometer	3319 independent reflections
Radiation source: fine-focus sealed tube	1736 reflections with *I* > 2σ(*I*)
Graphite monochromator	*R*_int_ = 0.076
ω scans	θ_max_ = 26.0°, θ_min_ = 2.1°
Absorption correction: multi-scan (*SADABS*; Bruker, 2007)	*h* = −15→15
*T*_min_ = 0.868, *T*_max_ = 0.931	*k* = −21→21
13580 measured reflections	*l* = −10→9

### Refinement

**Table:**

Refinement on *F*^2^	Primary atom site location: structure-invariant direct methods
Least-squares matrix: full	Secondary atom site location: difference Fourier map
*R*[*F*^2^ > 2σ(*F*^2^)] = 0.056	Hydrogen site location: inferred from neighbouring sites
*wR*(*F*^2^) = 0.140	H-atom parameters constrained
*S* = 1.00	*w* = 1/[σ^2^(*F*_o_^2^) + (0.047*P*)^2^ + 0.7016*P*] where *P* = (*F*_o_^2^ + 2*F*_c_^2^)/3
3319 reflections	(Δ/σ)_max_ < 0.001
218 parameters	Δρ_max_ = 0.42 e Å^−^^3^
0 restraints	Δρ_min_ = −0.33 e Å^−^^3^

### Special details

**Table:**

Geometry. All e.s.d.'s (except the e.s.d. in the dihedral angle between two l.s. planes) are estimated using the full covariance matrix. The cell e.s.d.'s are taken into account individually in the estimation of e.s.d.'s in distances, angles and torsion angles; correlations between e.s.d.'s in cell parameters are only used when they are defined by crystal symmetry. An approximate (isotropic) treatment of cell e.s.d.'s is used for estimating e.s.d.'s involving l.s. planes.
Refinement. Refinement of *F*^2^ against ALL reflections. The weighted *R*-factor *wR* and goodness of fit *S* are based on *F*^2^, conventional *R*-factors *R* are based on *F*, with *F* set to zero for negative *F*^2^. The threshold expression of *F*^2^ > σ(*F*^2^) is used only for calculating *R*-factors(gt) *etc*. and is not relevant to the choice of reflections for refinement. *R*-factors based on *F*^2^ are statistically about twice as large as those based on *F*, and *R*- factors based on ALL data will be even larger.

### Fractional atomic coordinates and isotropic or equivalent isotropic displacement parameters (Å^2^)

**Table:**

	*x*	*y*	*z*	*U*_iso_*/*U*_eq_	
C1	0.2793 (4)	0.0825 (2)	0.2790 (5)	0.0443 (10)	
C2	0.3546 (4)	0.0531 (2)	0.4159 (5)	0.0562 (12)	
H2	0.4291	0.0496	0.4103	0.067*	
C3	0.3216 (5)	0.0291 (3)	0.5591 (6)	0.0754 (16)	
H3	0.3733	0.0100	0.6489	0.090*	
C4	0.2111 (6)	0.0339 (3)	0.5677 (6)	0.0820 (18)	
H4	0.1879	0.0176	0.6635	0.098*	
C5	0.1353 (5)	0.0624 (3)	0.4352 (7)	0.0736 (15)	
H5	0.0609	0.0652	0.4421	0.088*	
C6	0.1678 (4)	0.0875 (2)	0.2903 (5)	0.0534 (11)	
C7	−0.0133 (4)	0.1564 (3)	0.1866 (7)	0.0877 (17)	
H7A	−0.0670	0.1177	0.2001	0.132*	
H7B	−0.0435	0.1892	0.0936	0.132*	
H7C	0.0046	0.1874	0.2861	0.132*	
C8	0.2436 (3)	0.1500 (2)	−0.0046 (5)	0.0456 (10)	
H8	0.2703	0.1445	−0.1090	0.055*	
C9	0.3205 (3)	0.1070 (2)	0.1305 (4)	0.0402 (9)	
C10	0.5527 (3)	0.1066 (2)	−0.0255 (5)	0.0460 (10)	
H10	0.5968	0.0836	0.0677	0.055*	
C11	0.6032 (3)	0.1273 (2)	−0.1647 (5)	0.0404 (9)	
C12	0.7137 (3)	0.1108 (2)	−0.1598 (5)	0.0511 (11)	
H12	0.7557	0.0875	−0.0653	0.061*	
C13	0.7630 (3)	0.1285 (2)	−0.2933 (6)	0.0551 (12)	
H13	0.8374	0.1172	−0.2886	0.066*	
C14	0.7011 (4)	0.1626 (2)	−0.4315 (5)	0.0508 (11)	
C15	0.5919 (4)	0.1809 (2)	−0.4393 (5)	0.0550 (11)	
H15	0.5509	0.2047	−0.5341	0.066*	
C16	0.5430 (3)	0.1640 (2)	−0.3062 (5)	0.0498 (11)	
H16	0.4691	0.1772	−0.3108	0.060*	
S1	0.10908 (9)	0.10838 (7)	−0.03153 (14)	0.0548 (3)	
N1	0.0871 (3)	0.1183 (2)	0.1564 (5)	0.0598 (10)	
N2	0.4212 (3)	0.09167 (19)	0.1225 (4)	0.0493 (9)	
N3	0.4512 (3)	0.11839 (19)	−0.0247 (4)	0.0504 (9)	
O1	0.0296 (2)	0.1538 (2)	−0.1431 (4)	0.0787 (10)	
O2	0.1256 (2)	0.02920 (17)	−0.0680 (3)	0.0573 (8)	
Cl1	0.23868 (10)	0.25052 (6)	0.04375 (17)	0.0757 (4)	
Cl2	0.76214 (12)	0.18282 (8)	−0.60081 (17)	0.0859 (5)	

### Atomic displacement parameters (Å^2^)

**Table:**

	*U*^11^	*U*^22^	*U*^33^	*U*^12^	*U*^13^	*U*^23^
C1	0.059 (3)	0.038 (2)	0.039 (2)	−0.009 (2)	0.015 (2)	−0.0026 (18)
C2	0.076 (3)	0.053 (3)	0.040 (2)	−0.012 (2)	0.013 (2)	−0.004 (2)
C3	0.120 (5)	0.067 (3)	0.039 (3)	−0.023 (3)	0.016 (3)	0.000 (2)
C4	0.151 (6)	0.056 (3)	0.051 (3)	−0.026 (4)	0.050 (4)	−0.009 (3)
C5	0.100 (4)	0.058 (3)	0.080 (4)	−0.017 (3)	0.057 (3)	−0.011 (3)
C6	0.072 (3)	0.038 (2)	0.057 (3)	−0.012 (2)	0.030 (3)	−0.011 (2)
C7	0.062 (3)	0.093 (4)	0.115 (4)	0.006 (3)	0.036 (3)	−0.032 (3)
C8	0.045 (3)	0.046 (2)	0.047 (2)	−0.0027 (19)	0.012 (2)	0.0048 (19)
C9	0.048 (3)	0.035 (2)	0.039 (2)	−0.006 (2)	0.0107 (19)	−0.0015 (18)
C10	0.048 (3)	0.044 (2)	0.046 (2)	0.008 (2)	0.009 (2)	0.0055 (19)
C11	0.036 (2)	0.040 (2)	0.045 (2)	−0.0033 (18)	0.0075 (19)	−0.0003 (18)
C12	0.041 (2)	0.053 (3)	0.058 (3)	0.005 (2)	0.005 (2)	0.001 (2)
C13	0.034 (2)	0.051 (3)	0.082 (3)	−0.001 (2)	0.016 (2)	−0.008 (2)
C14	0.057 (3)	0.040 (2)	0.061 (3)	−0.004 (2)	0.026 (2)	−0.001 (2)
C15	0.055 (3)	0.054 (3)	0.058 (3)	0.004 (2)	0.018 (2)	0.010 (2)
C16	0.037 (2)	0.055 (3)	0.058 (3)	0.000 (2)	0.012 (2)	0.004 (2)
S1	0.0448 (7)	0.0606 (7)	0.0593 (7)	−0.0038 (6)	0.0111 (5)	0.0011 (6)
N1	0.053 (2)	0.064 (2)	0.071 (2)	0.002 (2)	0.032 (2)	−0.003 (2)
N2	0.053 (2)	0.058 (2)	0.0383 (19)	0.0034 (18)	0.0119 (17)	0.0118 (16)
N3	0.045 (2)	0.063 (2)	0.045 (2)	0.0035 (18)	0.0116 (16)	0.0084 (18)
O1	0.0463 (19)	0.094 (3)	0.088 (2)	0.0067 (18)	−0.0040 (18)	0.017 (2)
O2	0.0593 (19)	0.0633 (19)	0.0498 (17)	−0.0074 (16)	0.0119 (15)	−0.0139 (15)
Cl1	0.0765 (9)	0.0449 (6)	0.1015 (10)	−0.0031 (6)	0.0077 (7)	0.0103 (6)
Cl2	0.1003 (11)	0.0762 (9)	0.1001 (10)	0.0026 (8)	0.0653 (9)	0.0138 (8)

### Geometric parameters (Å, º)

**Table:**

C1—C6	1.396 (6)	C9—N2	1.281 (5)
C1—C2	1.397 (5)	C10—N3	1.267 (5)
C1—C9	1.470 (5)	C10—C11	1.448 (5)
C2—C3	1.378 (6)	C10—H10	0.9300
C2—H2	0.9300	C11—C12	1.381 (5)
C3—C4	1.379 (7)	C11—C16	1.394 (5)
C3—H3	0.9300	C12—C13	1.387 (6)
C4—C5	1.371 (7)	C12—H12	0.9300
C4—H4	0.9300	C13—C14	1.363 (5)
C5—C6	1.394 (6)	C13—H13	0.9300
C5—H5	0.9300	C14—C15	1.369 (5)
C6—N1	1.425 (5)	C14—Cl2	1.739 (4)
C7—N1	1.464 (5)	C15—C16	1.378 (5)
C7—H7A	0.9600	C15—H15	0.9300
C7—H7B	0.9600	C16—H16	0.9300
C7—H7C	0.9600	S1—O2	1.417 (3)
C8—C9	1.498 (5)	S1—O1	1.428 (3)
C8—Cl1	1.776 (4)	S1—N1	1.623 (4)
C8—S1	1.776 (4)	N2—N3	1.406 (4)
C8—H8	0.9800		
			
C6—C1—C2	118.1 (4)	N3—C10—C11	123.0 (4)
C6—C1—C9	123.0 (4)	N3—C10—H10	118.5
C2—C1—C9	118.9 (4)	C11—C10—H10	118.5
C3—C2—C1	121.9 (5)	C12—C11—C16	118.2 (4)
C3—C2—H2	119.0	C12—C11—C10	120.2 (4)
C1—C2—H2	119.0	C16—C11—C10	121.6 (4)
C2—C3—C4	119.2 (5)	C11—C12—C13	121.1 (4)
C2—C3—H3	120.4	C11—C12—H12	119.4
C4—C3—H3	120.4	C13—C12—H12	119.4
C5—C4—C3	120.1 (5)	C14—C13—C12	119.2 (4)
C5—C4—H4	119.9	C14—C13—H13	120.4
C3—C4—H4	119.9	C12—C13—H13	120.4
C4—C5—C6	121.2 (5)	C13—C14—C15	121.1 (4)
C4—C5—H5	119.4	C13—C14—Cl2	119.2 (3)
C6—C5—H5	119.4	C15—C14—Cl2	119.6 (4)
C5—C6—C1	119.5 (5)	C14—C15—C16	119.7 (4)
C5—C6—N1	119.6 (4)	C14—C15—H15	120.1
C1—C6—N1	120.9 (4)	C16—C15—H15	120.1
N1—C7—H7A	109.5	C15—C16—C11	120.6 (4)
N1—C7—H7B	109.5	C15—C16—H16	119.7
H7A—C7—H7B	109.5	C11—C16—H16	119.7
N1—C7—H7C	109.5	O2—S1—O1	119.9 (2)
H7A—C7—H7C	109.5	O2—S1—N1	111.05 (18)
H7B—C7—H7C	109.5	O1—S1—N1	108.9 (2)
C9—C8—Cl1	111.1 (3)	O2—S1—C8	104.09 (18)
C9—C8—S1	109.1 (3)	O1—S1—C8	111.11 (19)
Cl1—C8—S1	110.3 (2)	N1—S1—C8	99.76 (19)
C9—C8—H8	108.7	C6—N1—C7	121.2 (4)
Cl1—C8—H8	108.7	C6—N1—S1	117.8 (3)
S1—C8—H8	108.7	C7—N1—S1	121.0 (3)
N2—C9—C1	118.8 (4)	C9—N2—N3	113.6 (3)
N2—C9—C8	122.5 (3)	C10—N3—N2	112.4 (3)
C1—C9—C8	118.6 (4)		
			
C6—C1—C2—C3	0.1 (6)	C13—C14—C15—C16	−0.6 (6)
C9—C1—C2—C3	−180.0 (4)	Cl2—C14—C15—C16	179.1 (3)
C1—C2—C3—C4	0.3 (7)	C14—C15—C16—C11	−1.0 (6)
C2—C3—C4—C5	−0.3 (7)	C12—C11—C16—C15	2.1 (6)
C3—C4—C5—C6	−0.3 (7)	C10—C11—C16—C15	−178.0 (4)
C4—C5—C6—C1	0.7 (6)	C9—C8—S1—O2	−57.3 (3)
C4—C5—C6—N1	−178.7 (4)	Cl1—C8—S1—O2	−179.68 (19)
C2—C1—C6—C5	−0.6 (6)	C9—C8—S1—O1	172.3 (3)
C9—C1—C6—C5	179.5 (4)	Cl1—C8—S1—O1	49.9 (3)
C2—C1—C6—N1	178.8 (3)	C9—C8—S1—N1	57.4 (3)
C9—C1—C6—N1	−1.1 (6)	Cl1—C8—S1—N1	−64.9 (2)
C6—C1—C9—N2	−171.5 (4)	C5—C6—N1—C7	27.6 (6)
C2—C1—C9—N2	8.6 (5)	C1—C6—N1—C7	−151.8 (4)
C6—C1—C9—C8	9.5 (5)	C5—C6—N1—S1	−151.3 (3)
C2—C1—C9—C8	−170.4 (3)	C1—C6—N1—S1	29.3 (5)
Cl1—C8—C9—N2	−96.8 (4)	O2—S1—N1—C6	55.3 (3)
S1—C8—C9—N2	141.3 (3)	O1—S1—N1—C6	−170.5 (3)
Cl1—C8—C9—C1	82.1 (4)	C8—S1—N1—C6	−54.0 (3)
S1—C8—C9—C1	−39.7 (4)	O2—S1—N1—C7	−123.6 (4)
N3—C10—C11—C12	−178.1 (4)	O1—S1—N1—C7	10.6 (4)
N3—C10—C11—C16	1.9 (6)	C8—S1—N1—C7	127.1 (4)
C16—C11—C12—C13	−1.6 (6)	C1—C9—N2—N3	−179.5 (3)
C10—C11—C12—C13	178.5 (4)	C8—C9—N2—N3	−0.6 (5)
C11—C12—C13—C14	0.0 (6)	C11—C10—N3—N2	178.6 (3)
C12—C13—C14—C15	1.2 (6)	C9—N2—N3—C10	176.2 (4)
C12—C13—C14—Cl2	−178.6 (3)		

### Hydrogen-bond geometry (Å, º)

**Table:**

*D*—H···*A*	*D*—H	H···*A*	*D*···*A*	*D*—H···*A*
C4—H4···O2^i^	0.93	2.48	3.356 (6)	158
C12—H12···O2^ii^	0.93	2.59	3.419 (5)	149
C13—H13···O1^iii^	0.93	2.50	3.293 (5)	143

Symmetry codes: (i) *x*, *y*, *z*+1; (ii) −*x*+1, −*y*, −*z*; (iii) *x*+1, *y*, *z*.
